# Decoding neurodegeneration one cell at a time

**DOI:** 10.1172/JCI199841

**Published:** 2026-03-16

**Authors:** Olivia Gautier, Thao P. Nguyen, Aaron D. Gitler

**Affiliations:** 1 Department of Genetics and; 2 Stanford Neurosciences Graduate Program, Stanford University School of Medicine, Stanford, California, USA.

## Abstract

Neurodegenerative diseases are characterized by protein misfolding and the selective vulnerability of specific neuronal subtypes. This selective vulnerability presents a paradox; most neurodegenerative disease genes are expressed broadly throughout the brain, and some ubiquitously, but only certain types of neurons are lost while others are resistant. The molecular basis for selective neuronal vulnerability has remained a mystery, but recent genomics technological innovations are starting to provide mechanistic insights. Here, we review how single-cell genomics techniques — single-cell transcriptomics, single-cell epigenomics, and spatial transcriptomics — advance our molecular understanding of selective vulnerability and neurodegeneration across Alzheimer disease, Parkinson disease, amyotrophic lateral sclerosis, frontotemporal dementia, and Huntington disease. Together, these approaches reveal the cell types affected in disease, define disease-associated molecular states, nominate candidate determinants of vulnerability and degeneration, and situate degenerating neurons within their local tissue context. Continued development and application of these techniques, including single-cell perturbation screens, will expand descriptive atlases of relevant cell types in health and disease and identify causal mechanisms, revealing the molecular basis of vulnerability and degeneration and informing therapeutic development.

## Introduction

**Figure 1:**
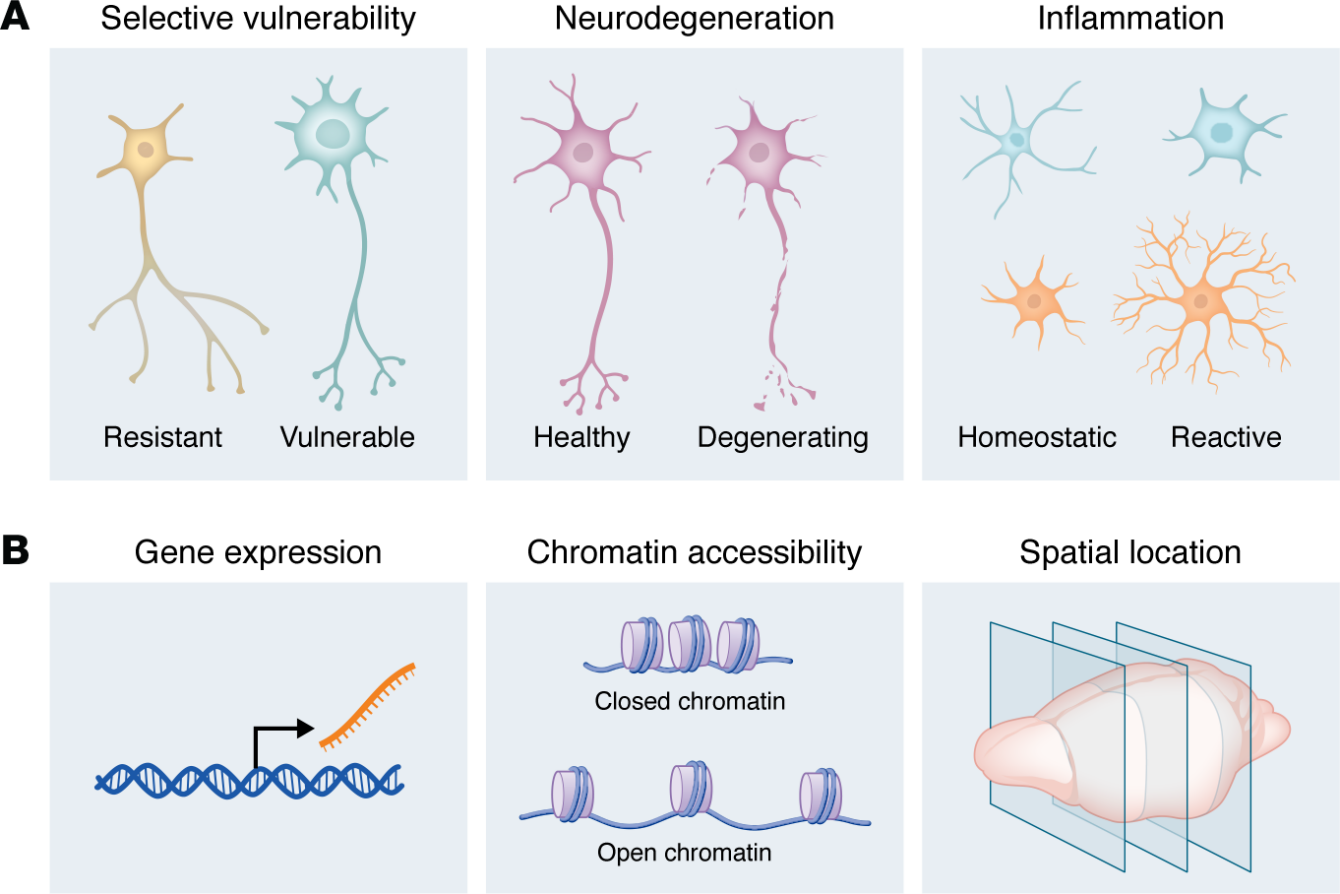
Single-cell comparisons and readouts for neurodegenerative diseases. (**A**) Comparative conditions in single-cell studies of neurodegeneration. Left: A resistant versus vulnerable neuronal subtype for a given neurodegenerative disease. Middle: A healthy versus degenerating neuron of the same cell type. Right: Homeostatic versus reactive glia (e.g., microglia and astrocytes) in disease. Contrasting these conditions with single-cell datasets reveals molecular correlates of selective vulnerability, degeneration, and glial responses. (**B**) Single-cell readouts that capture complementary biology. Left: Gene expression — scRNA-seq profiles define disease-associated transcriptional changes and cell states. Middle: Chromatin accessibility — scATAC-seq maps regulatory elements and transcription factor motif accessibility, which may promote disease-associated states. Right: Spatial context — spatial transcriptomics localizes cell states in situ, resolving the cellular neighbors of degenerating neurons. Together, these readouts help relate cell identity, cell state, regulatory factors, and spatial context, offering a more integrated view of disease biology.

Neurodegenerative diseases remain one of the most urgent challenges in modern medicine, owing to their high prevalence and the lack of effective disease-modifying therapies. These diseases share core pathological hallmarks, including misfolding and aggregation of proteins such as amyloid-β, tau, α-synuclein, and TDP-43, often accompanied by chronic neuroinflammation (1, 2). Despite these shared features, each disorder produces distinct clinical manifestations through selective vulnerability; specific brain regions and neuronal subtypes are disproportionately affected by pathogenic processes and subsequent cell death (3–5). Why do only certain neurons succumb to disease? How do non-neuronal cell types either protect neurons or amplify cytotoxic processes? How do different genetic risk factors act within specific cellular contexts to influence disease risk? These key questions have yet to be fully addressed (Figure 1A).

Early sequencing efforts to study neurodegenerative disease relied on bulk tissue analyses from mouse models and human postmortem samples. These have been instrumental in identifying broad molecular changes, but bulk methods yield a single, averaged molecular signature from millions of cells within a sample. Such averaging masks the intricate cellular heterogeneity of the central nervous system (CNS), comprising billions of cells across diverse types, and obscures cell-type-specific changes thought to drive pathogenesis.

Recent advances in genomics have yielded sequencing-based methods capable of capturing molecular features at single-cell resolution. These include single-cell and single-nucleus RNA-seq (sc/snRNA-seq) to profile the transcriptomes of individual cells (6–12), single-cell and single-nucleus assays for transposase-accessible chromatin using sequencing (sc/snATAC-seq) to map chromatin accessibility (13, 14), and spatial transcriptomics (15–21), which integrates single-cell transcriptomics with spatial information to study cells within their native tissue niches (Figure 1B). Additionally, single-cell assays can be coupled with forward genetic screens to map the effects of genetic perturbations on cellular transcriptomes (e.g., Perturb-seq, ECCITE-seq, CROP-seq), epigenomes (e.g., CRISPR-sciATAC, Perturb-ATAC, Spear-ATAC), and spatial locations (e.g., Perturb-Multimodal) (22–31).

**Figure 2:**
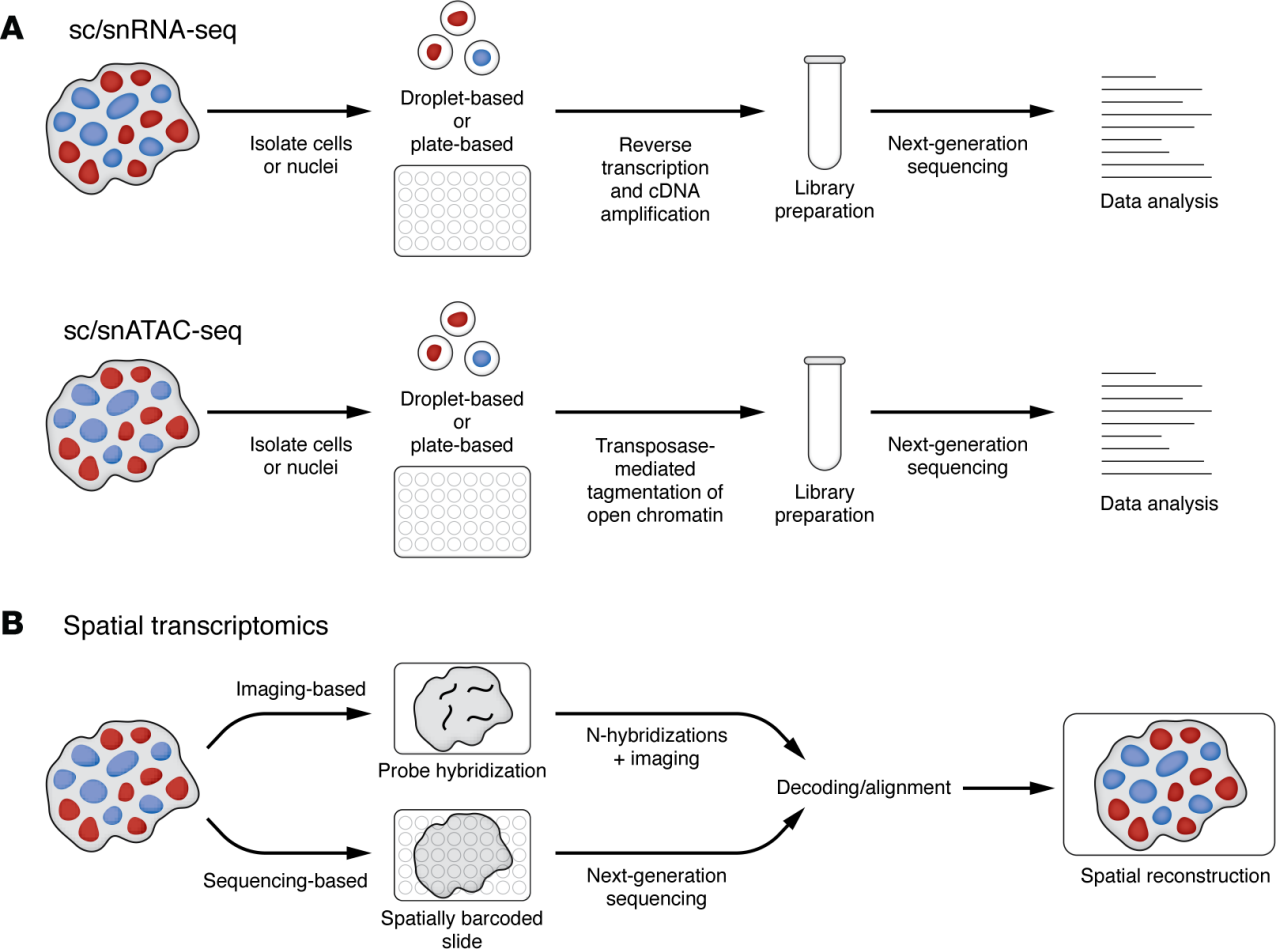
Overview of single-cell and spatial transcriptomic sequencing workflows. (**A**) Typical sc/snRNA-seq and sc/snATAC-seq workflows. Individual cells or nuclei are isolated from fresh, frozen, or fixed tissues or cultured cells. Depending on the platform, they are either singly encapsulated in barcoded droplets or separated into individual wells for reverse transcription and cDNA amplification (sc/snRNA-seq) or transposase-mediated tagmentation of open chromatin (sc/snATAC-seq), followed by library preparation and next-generation sequencing. (**B**) Spatial transcriptomics approaches. Imaging-based approaches (e.g., MERFISH, STARmap, 10x Genomics Xenium) use multiplexed probe hybridization and fluorescence imaging to detect transcripts at nearly single-cell resolution, whereas sequencing-based approaches (e.g., Nanostring’s GeoMx, 10x Genomics Visium) capture RNA on spatially barcoded slides followed by sequencing.

Typical sc/snRNA-seq and sc/snATAC-seq workflows involve isolating individual cells or nuclei from tissues or cultured cells, which can be fresh, frozen, or fixed (Figure 2A). Depending on the platform, cells or nuclei are then singly encapsulated in barcoded droplets or separated into individual wells in multiwell plates for reverse transcription and cDNA amplification (sc/snRNA-seq) or transposase-mediated tagmentation (a process that simultaneously fragments DNA and attaches short adapter sequences to the fragments) of open chromatin (sc/snATAC-seq), followed by library preparation and next-generation sequencing.

How do we decide whether we should choose single-cell or single-nucleus sequencing? This depends on sample types and biological applications. Single-cell sequencing is typically applied to fresh, readily dissociable tissues or cultured cells to study intact cell populations. Because it captures both cytoplasmic and nuclear transcripts, scRNA-seq provides a comprehensive view of cellular gene expression. However, tissue dissociation can induce stress-related transcriptional artifacts and introduce substantial cell-type bias. Large or fragile neurons are often lost during dissociation, whereas smaller cell types, such as astrocytes and oligodendrocytes, tend to be overrepresented. In contrast, single-nucleus sequencing is commonly used for frozen samples or for tissues that are difficult to dissociate, including the brain and spinal cord. Although fresh or fresh-frozen samples are typically used, snRNA-seq is compatible with formalin-fixed, paraffin-embedded (FFPE) samples, enabling the analysis of archived human specimens. A key limitation is that snRNA-seq does not capture cytoplasmic transcripts and is therefore biased toward nuclear, often premature, mRNA species.

Spatial transcriptomics does not require tissue dissociation and enables examination of cellular transcriptomes within their native tissue niches. Some spatial transcriptomic technologies are now compatible with FFPE samples, allowing analyses of preserved clinical specimens along with fixed-frozen and fresh-frozen samples. These technologies can be broadly classified into two main categories: imaging-based and sequencing-based (Figure 2B). Imaging-based approaches, like multiplexed error-robust fluorescence in situ hybridization (MERFISH), spatially resolved transcript amplicon readout mapping (STARmap), and 10x Genomics Xenium, rely on probe hybridization and multiplexed imaging to detect and visualize transcripts at high spatial resolution, often achieving single-cell or even subcellular resolution (17, 18). Although whole-transcriptome measurements are possible, MERFISH typically targets predefined gene panels due to the constraints of iterative hybridization and imaging. In contrast, sequencing-based approaches, including NanoString GeoMx and 10x Genomics Visium, capture RNA on spatially barcoded tissue slides or nanobeads followed by next-generation sequencing. These methods generally recover a broader range of transcripts than imaging-based approaches but, in most cases, do not yet achieve true single-cell resolution. Instead, they measure gene expression within spatial “spots” that encompass multiple cells and therefore rely on computational deconvolution to infer cell-type composition. Newer spatial transcriptomic methods, like spatial enhanced resolution omics sequencing (Stereo-seq) and reverse-padlock amplicon-encoding fluorescence in situ hybridization (RAEFISH), are approaching single-cell and single-molecule resolution (19–21).

In this Review, we summarize recent advances and applications of single-cell genomics approaches to study neurodegenerative disorders, including Alzheimer disease (AD), Parkinson disease (PD), amyotrophic lateral sclerosis (ALS), frontotemporal dementia (FTD), and Huntington disease (HD). We focus on how these approaches provide insight into the unique vulnerabilities of specific neuronal populations, define novel disease-associated cellular states, and reveal contributions of non-neuronal cells to disease pathogenesis. We then look to the future, envisioning how these technologies will empower genetic screens to uncover modifiers of neurodegeneration and new therapeutic targets.

## Single-cell genomic approaches in neurodegenerative disorders

### AD.

AD, the most common neurodegenerative disorder, typically progresses from mild cognitive impairment to severe dementia. Neuropathologically, AD is defined by amyloid plaques and tau tangles and follows a stereotyped trajectory in which entorhinal, hippocampal, and neocortical neurons are selectively lost, alongside vulnerable subcortical and interneuron populations (32, 33). Single-cell technologies now allow these patterns of selective vulnerability to be explored with molecular resolution.

Early snRNA-seq studies provided the first atlases of the AD brain, revealing cell-type-specific changes across neurons, microglia, astrocytes, oligodendrocyte lineages, and endothelial cells (34–36). As datasets grew, neuronal subtypes with distinct molecular signatures of vulnerability were identified. Leng et al. showed that *RORB*^+^ excitatory neurons in entorhinal cortex layer 2 are selectively vulnerable in AD, accompanied by the early downregulation of synaptic genes prior to cell death (37). Building on this, Consens et al., Cain et al., and Mathys et al. reported selective loss of *SST*^+^ interneurons in the frontal cortex (38–40). Gabitto et al. extended these findings by showing in the middle temporal gyrus that *SST*^+^ interneurons are lost early in disease, while excitatory neurons and *PVALB*^+^ and *VIP*^+^ inhibitory neuron subtypes decline later, clarifying the temporal sequence of neuronal vulnerability in this brain region (41). Rexach et al. additionally found selective depletion of a layer 5 intratelencephalic excitatory neuron population marked by expression of *RORB* and *NEFM* in AD (42). As a complement to studies with postmortem samples, Gazestani et al. analyzed surgical biopsy tissue from living individuals with varying degrees of AD pathology and uncovered a hyperactive state preceding excitatory neuron loss (43). A meta-analysis incorporating postmortem snRNA-seq datasets revealed depletion of *NDNF-PROX1*^+^ layer 1 interneurons and *LINC00507-COL5A2*^+^ layer 2/3 telencephalic neurons in samples with mild amyloid pathology (43). Together, these studies delineate the transcriptomic signatures of selectively vulnerable neuronal subtypes in AD and chart their molecular changes with disease.

Single-cell studies have made it clear that non-neuronal populations, such as microglia, are integral to AD pathogenesis. Prater et al. reported increased abundance of a microglia subcluster enriched for lysosomal/vesicular function, interferon regulatory factor, and inflammasome activation genes in disease, along with an AD-specific subcluster within homeostatic-marker-expressing microglia (44). Rexach et al. further found an AD-specific amyloid-associated microglia cluster marked by *ITM2B*, which may be protective (42). Gabitto et al. showed that inflammatory microglia appear early in AD in the middle temporal gyrus, preceding loss of excitatory neurons, highlighting glia as potential early drivers of disease (41).

Astrocytes also undergo prominent AD-associated changes. Serrano-Pozo et al. found that the proportion of homeostatic, intermediate, and reactive astrocytes varied across regions, while other subclusters changed with AD pathology stage (45). Consistent with this, Gabitto et al. reported early reactive astrocytes in the middle temporal gyrus that appear before excitatory neuron loss (41). Rare cases of protection from AD pathology highlight how glial response may mitigate disease. For example, Almeida et al. reported a familial AD gene mutation carrier who also harbored two copies of a protective genetic mutation (46). She remained cognitively intact into her 70s and, after her death, snRNA-seq revealed astrocytic *LRP1* upregulation, consistent with enhanced tau uptake and clearance and a protective glial response (46).

Additionally, other non-neuronal cell types are affected in AD. Gabitto et al. observed early loss of oligodendrocytes in disease, accompanied by a remyelination program in oligodendrocyte precursor cells (41). Vascular cells exhibit AD-associated transcriptional changes; Yang et al. generated a molecular atlas of the human brain vasculature and identified AD-associated expression changes consistent with dysregulated blood flow (47), and Sun et al. reported disease-associated transcriptional changes in cerebrovasculature cells (48). Both studies implicated AD genome-wide association study (GWAS) variants in vasculature cell types, broadening genetic risk beyond microglia and neurons (47, 48).

snATAC-seq and multiomic studies are defining the epigenetic landscapes of brain cell types and drivers of AD-associated state changes. Early scATAC-seq studies showed that AD GWAS variants are enriched in microglia-accessible chromatin, linking genetic risk to cell-type-specific regulatory elements (49). Liu et al. extended this framework to multiregion single-nucleus epigenomic and multiomic profiling across AD pathology progression (50). They found widespread epigenomic relaxation and information loss with advancing pathology, alongside preserved epigenomic stability in cognitively resilient individuals (50). Single-cell epigenomic and multiomic studies have identified disease-relevant transcription factors in AD by linking cell-type-specific chromatin accessibility and motif enrichment to AD risk loci and disease-associated states in glial cells, implicating these factors as potential regulators of cell state transitions (41, 42, 51–54).

Spatially resolved approaches have deepened our understanding of how these states are organized in tissue. Using STARmap with protein localization and unlimited sequencing (STARmap PLUS) in a mouse model of AD, Zeng et al. found a core-shell structure; disease-associated microglia closely contact amyloid-β plaques at the core, while diseased astrocytes and oligodendrocyte precursor cells populate outer shells (55). Hyperphosphorylated tau appears predominantly in CA1 excitatory neurons and tracks with local enrichment of oligodendrocyte subtypes (55). Gabitto et al. leveraged MERFISH spatial transcriptomics to validate and refine single-cell findings in AD cortex, confirming loss of vulnerable neurons and adding spatial context that dissociated snRNA-seq alone could not provide (41).

**Figure 3:**
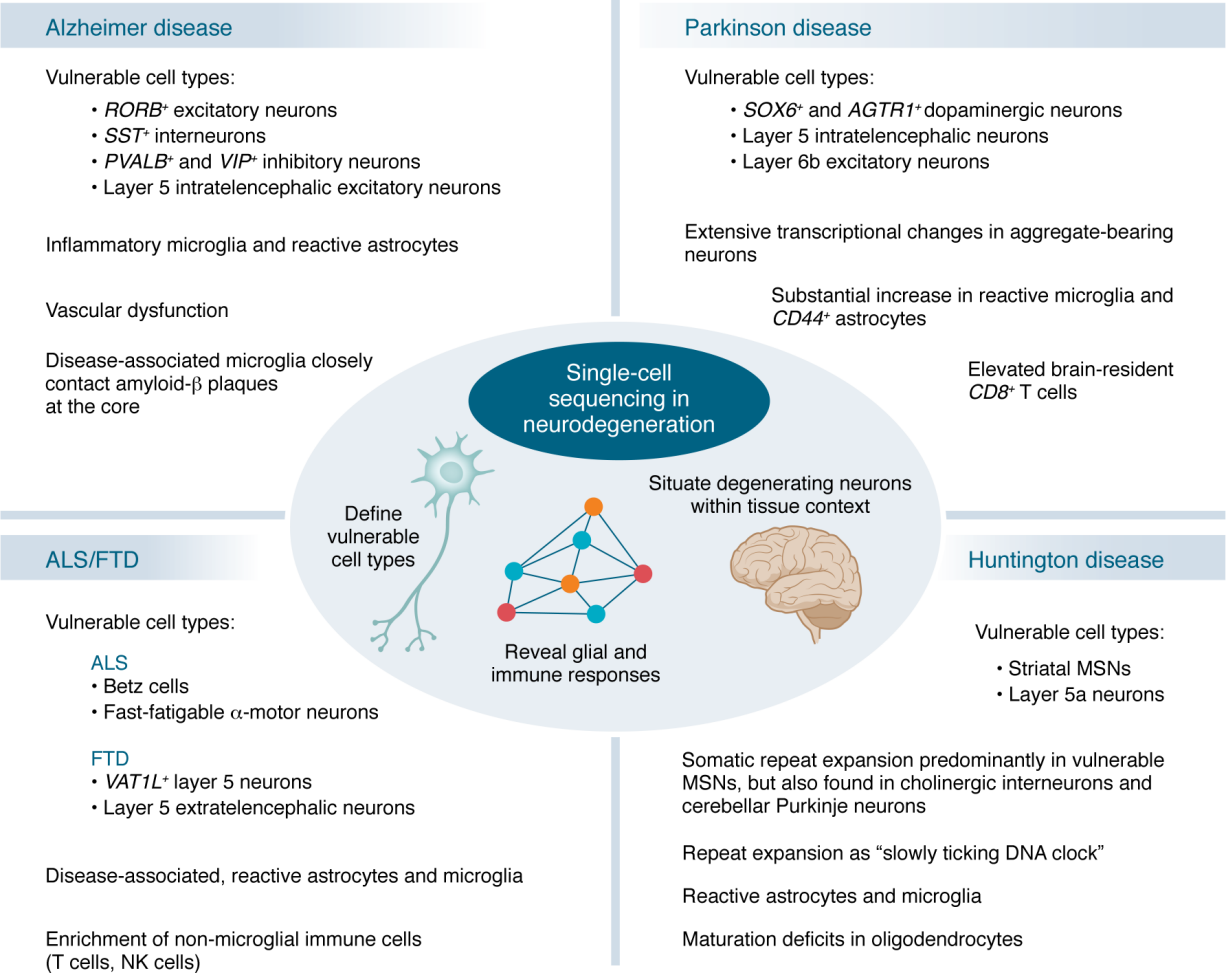
Insights into neurodegenerative disease mechanisms from single-cell analyses. Major findings from single-cell studies for Alzheimer disease, Parkinson disease, amyotrophic lateral sclerosis, frontotemporal dementia, and Huntington disease.

Together, these studies identify selectively vulnerable neurons (e.g., *RORB*^+^ excitatory, *SST*^+^ interneurons) as well as disease-associated transcriptional changes in glial and vascular cells. Multiomic analyses implicate transcriptional regulators of these states, while spatial transcriptomics reveals how these cell types are organized around AD pathology (Figure 3).

### PD.

PD is characterized by classic motor symptoms such as bradykinesia and tremor, as well as non-motor symptoms such as depression, REM sleep behavior disorder, and orthostatic hypotension (56, 57). A neuropathological hallmark of PD is the presence of α-synuclein aggregates known as Lewy bodies and Lewy neurites, accompanied by the selective loss of dopaminergic neurons (DaNs) in the substantia nigra pars compacta (SNpc) (57, 58). The mechanisms underlying selective neuronal vulnerability in PD have been challenging to dissect, but early single-cell studies confirmed the loss of these neurons and, in doing so, began to unravel the molecular features that confer their vulnerability.

Major advancements in defining vulnerable neuronal populations in PD came from single-cell transcriptomic profiling of human postmortem tissue. Kamath et al. used single-cell genomic profiling of DaNs from patients with PD and age-matched controls, identifying a highly susceptible subpopulation marked by *AGTR1* expression, predominantly localized to the ventral tier of the SNpc (59). This population exhibited strong upregulation of *TP53-* and *NR2F2-*regulated genes, implicating these molecular pathways in neurodegeneration (59). Importantly, this population was preferentially enriched for genetic PD risk factors, pointing to cell-intrinsic mechanisms as key contributors of DaN selective vulnerability (59).

Smajić et al. performed snRNA-seq on postmortem midbrain tissue from patients with idiopathic PD and controls, identifying a neuronal cluster derived almost exclusively from PD samples (60). These cells were characterized by high expression of *CADPS2*, a calcium-dependent activator of secretion, and shared key transcriptional features with DaNs (60). However, they exhibited reduced expression of *TH* (tyrosine hydroxylase) and increased expression of *TIAM1*, a regulator of DaN differentiation, suggesting that they may represent a transitional or degenerating state (60).

Vulnerability to α-synuclein pathology and cell death in PD extends beyond DaNs. Zeng et al. constructed an extensive transcriptional atlas by combining published snRNA-seq datasets from patients with PD and controls, revealing widespread neuronal loss affecting multiple midbrain populations, including glutamatergic, GABAergic, and dopaminergic neurons (61). This comprehensive approach identified necroptosis as a key pathway underlying neuronal degeneration, with upregulation of necroptosis-related genes observed across all three vulnerable neuronal populations (61). Complementing these findings, Martirosyan et al. analyzed large-scale transcriptome data and found that both depleted GABAergic and dopaminergic neuronal subpopulations shared molecular signatures related to protein misfolding stress, oxidative damage, metabolic dysfunction, and disrupted iron homeostasis (62). This convergence of pathological pathways across different neuronal types suggests common vulnerability mechanisms underlying PD pathogenesis.

Beyond the midbrain, single-cell genomics has revealed that cortical regions also exhibit vulnerability to PD pathology. Using spatial transcriptomics to compare whole-transcriptome signatures of cortical neurons with and without α-synuclein pathology, Goralski et al. identified specific classes of excitatory neurons — layer 5 intratelencephalic and layer 6b neurons — that are particularly susceptible to developing Lewy pathology in both patients with PD and mouse models (63). They also discovered conserved gene expression changes in aggregate-bearing neurons, which they termed the Lewy-associated molecular dysfunction from aggregates (LAMDA) signature, providing insight into the molecular consequences of protein aggregation across distinct brain regions (63).

Single-cell genomics has also contributed to shifting the understanding of PD from a neuron-centric model to a complex system involving widespread changes across non-neuronal cells, including glia and brain-resident T cells. Studies using snRNA-seq and spatial transcriptomics in human postmortem tissues have identified specific dysregulations in astrocytes and microglia, including altered cytokine signaling and the adoption of proinflammatory states, alongside a notable enrichment of T cells in affected brain regions (60, 64, 65). These findings highlight the role of spatially restricted neuroinflammatory niches formed through interactions among multiple glial and immune cell types, which may serve as focal sites of neurodegeneration (64, 65).

Collectively, single-cell approaches provide a comprehensive picture of the specific neuronal populations vulnerable in PD, ranging from DaNs in the substantia nigra to layer 5 intratelencephalic and layer 6b neurons in the cortex. These methods also reveal widespread responses and contributions from multiple brain cell types, including astrocytes, microglia, and brain-resident T cells to disease pathogenesis (Figure 3).

### ALS and FTD.

ALS presents with progressive muscle weakness, paralysis, and ultimately respiratory failure due to degeneration of upper motor neurons in the motor cortex and lower motor neurons in the brainstem and spinal cord (66). Pathology most often involves nuclear loss and cytoplasmic aggregation of TDP-43, with rarer familial forms showing FUS or SOD1 inclusions. Vulnerability is selective; Betz cells and fast-fatigable α-motor neurons are especially affected, whereas γ- and visceral motor neurons in the spinal cord are largely spared (67). FTD manifests with behavioral and/or language decline from frontal and temporal lobe degeneration (68). Most cases show inclusions of TDP-43, TAF15, or tau (69). The behavioral variant of FTD (bvFTD) targets the anterior cingulate and frontoinsular cortex, where von Economo neurons (VENs) and fork cells (layer 5 projection neurons) are highly vulnerable, alongside subsets of layer 2/3 excitatory neurons (70, 71). Importantly, ALS and FTD share genetic risk factors, including a hexanucleotide repeat expansion in *C9ORF72*, helping explain their clinical overlap in some patients and common TDP-43 pathology (66, 72).

scRNA-seq and snRNA-seq have progressively defined which cell types are most affected and how. Starting in the adult mouse spinal cord, Blum et al. and Alkaslasi et al. profiled lower motor neuron subtypes, providing reference atlases of α-, γ-, and visceral motor neurons and their transcriptional diversity (73, 74). Extending into the human spinal cord, Gautier et al. sequenced a small set of lower motor neurons, including α and γ subtypes (75). This work demonstrates feasibility but also underscores the rarity of lower motor neurons as a challenge to robustly characterizing their heterogeneity in health and disease.

Turning to human cortex, Li et al. used snRNA-seq to show that in *C9ORF72* expansion carriers with ALS, there is notable transcriptional dysregulation in layer 2/3 and 5/6 excitatory neurons, including an increase in proteostasis genes and a decrease in neuronal function genes, whereas fewer high-quality neurons were recovered in C9-FTD (76). Gittings et al. applied snRNA-seq to frontal and occipital cortices from *C9ORF72* patients spanning the ALS/FTD disease spectrum and detected cryptic exons in TDP-43 target genes with single-cell resolution (77). *STMN2* and *KALRN* cryptic exon-containing transcripts were enriched in excitatory neurons, particularly in C9-FTD, with the highest proportion in layer 5 extratelencephalic neurons — a population that is transcriptionally similar to VENs and fork cells (77). Scaling further, Pineda et al. used snRNA-seq to profile ALS motor cortex and FTD prefrontal cortex and found that Betz cells and VENs are nearly indistinguishable transcriptionally and belong to a broader population of *VAT1L*^+^ layer 5 neurons that are enriched for ALS/FTD-linked genes and exhibit dysregulation of primary cilia and axoneme-related genes in disease (78). Additional work suggests that this *VAT1L*^+^ population (also marked by *GABRQ*) is depleted in ALS and FTD (78, 79). Pineda and colleagues also identified a layer 3/5 *SCN4B*^+^ excitatory neuron population in the motor cortex, which may be disease relevant and warrants further investigation (78). Limone et al. applied snRNA-seq to ALS and control cortex samples and found that *THY1*^+^ extratelencephalic neurons have higher expression of ALS/FTD risk genes than other cortical neurons, even in controls, potentially contributing to their inherent susceptibility in disease (80). In ALS, extratelencephalic neurons exhibited an induction of genes involved in protein homeostasis and stress responses (80). Furthermore, in bvFTD, Rexach et al. found selective vulnerability of layer 2/3 intratelencephalic neurons as well as enrichment of a layer 2/3 excitatory neuron cluster in the insular cortex marked by increased expression of multiple bvFTD/ALS risk genes (42).

Besides neurons, across ALS/FTD, astrocytes, oligodendrocyte lineage cells, and microglia show prominent disease-associated shifts, including astrocyte activation and cytoskeletal changes, downregulation of oligodendrocyte genes involved in myelination, differentiation, and axonal support, and loss of homeostatic programs accompanied by induction of reactive, interferon-responsive, and endolysosomal stress pathways (76, 78, 80, 81). Endothelial cells downregulate tight and adherens junction genes consistent with blood-brain-barrier dysfunction, alongside disease-associated changes in immune cell composition, including depletion of potentially protective microglial subtypes and enrichment of nonmicroglial immune cells like natural killer and T cells in affected regions (78, 81).

Li et al. generated a cortical snATAC-seq resource, defining cell-type-specific chromatin landscapes in control, ALS, and FTD conditions (76). Even though differentially accessible regions between the control and disease conditions were limited, comparison with their snRNA-seq data revealed concordant gene-activity and expression changes in glia, consistent with epigenetic remodeling in disease (76). Rexach et al. used snATAC-seq to identify transcription factor drivers of disease-associated cell states across dementias (42). In bvFTD, transcription factor footprinting showed increased RORB binding in vulnerable layer 2/3 excitatory neurons from insula, complementing the upregulation of *RORB* expression in these neurons in their snRNA-seq data (42). Most RORB target genes were downregulated in disease, suggesting a potential dampening of neuroprotective pathways (42). Rexach and colleagues further found an increase in chromatin accessibility at RUNX1 binding sites as well as upregulation of IKZF1 regulon target genes in bvFTD microglia, implicating these transcription factors in reactive microglial states (42).

A coherent picture emerges: long-range projection neurons, including Betz cells in ALS and VENs in FTD, are especially vulnerable, showing shared disease-associated transcriptional signatures and enrichment of ALS/FTD risk genes. Lower motor neurons remain central but difficult to interrogate because of their rarity. Glial populations exhibit loss of homeostatic functions and adoption of disease-associated or reactive states that may contribute to disease (Figure 3).

### HD.

HD is a monogenic neurodegenerative disorder caused by a CAG-repeat expansion in exon 1 of the huntingtin (*HTT*) gene (82, 83). Although *HTT* is ubiquitously expressed, the disease is characterized by preferential degeneration of medium spiny neurons (MSNs) in the striatum and deep-layer pyramidal neurons in the cortex, which are critical for motor control and cognitive function (84, 85). Burns et al. integrated spatial transcriptomics with snRNA-seq in the rapidly progressing R6/2 HD mouse model and found that the most vulnerable populations — MSNs in the striatum and excitatory neurons in the cortex — exhibit marked gene dysregulation as early as postnatal day 0 (86). These changes include early metabolic and maturation defects in the caudate putamen and dysregulation of kinesin-mediated migratory pathways in the cortex (86). However, because the R6/2 model expresses only mutant HTT (mHTT) exon 1, it lacks many of the MSN-selective pathologies found in mouse models expressing full-length mHTT (87, 88). More recent work using the full-length knockin mouse model has demonstrated robust, MSN-selective transcriptomic dysregulation and nuclear HTT aggregation that more faithfully recapitulates human pathology (88).

Single-cell analyses have revealed that within the striatum, the primary affected region, neuronal vulnerability is not uniform (86). Matsushima et al. showed that among striatal projection neurons (SPNs), striosomal SPNs are more vulnerable than matrix SPNs, and within the striosomes, D2 neurons are more affected than D1 neurons (89). As the disease progresses, these distinct striatal cell types begin to lose their unique molecular identities, blurring the distinction between striosomes and the matrix (89). Complementing these observations, Burns et al. used spatial and single-nucleus analyses of the R6/2 mice to show that changes in MSN identity genes are present at birth and intensify over time, whereas other cell types lose identity later (86). Thus, HD pathogenesis involves not only cell death but also progressive, regionally patterned collapse of cellular identity (86).

An important insight from single-cell genomics is the revelation that the inherited CAG-repeat expansion seems to function as a “slowly ticking DNA clock” rather than as an immediately toxic mutation, which may help explain why HD symptoms take decades to manifest. Handsaker et al. developed an innovative approach to simultaneously measure CAG-repeat length and genome-wide RNA expression at single-cell resolution (90). Applying this approach to postmortem samples from 50 patients with HD and 53 controls, they found that the CAG repeat tract expands gradually over decades in SPNs, growing at a rate of less than one repeat per year during the first two decades of life (90). Once a cell’s repeat tract reaches a critical “tipping point” of approximately 80 CAGs, the expansion rate accelerates dramatically, and the tract can reach 150 CAGs within just a few years (90). Once this threshold of approximately 150 repeats is breached, the cell’s transcriptome becomes profoundly dysregulated, ultimately leading to rapid cell death (90).

Using the full-length mHTT mouse model, Wang et al. independently identified a similar pathological threshold of approximately 150 CAG repeats (88). Importantly, they demonstrated that mismatch repair (MMR) genes *Msh3* and *Pms1* drive rapid, MSN-selective CAG-repeat expansion, leading to transcriptional dysregulation and mHTT aggregation (88). Knockout of *Msh3* in HD mice reduced *mHtt* repeat expansion in MSNs, ameliorated transcriptional dysregulation, and corrected several behavioral deficits (88). Thus, modulating *MSH3/PMS1* and their encoded MMR complexes may be a promising avenue for mitigating HD pathology and potentially other repeat expansion disorders.

Using snRNA-seq in brains from patients with HD, Handsaker et al. found that SPNs exhibit the most pronounced somatic expansion of the HD-causing allele compared with other cell types, which may explain their unique vulnerability (90). Using fluorescence-activated nuclear sorting of human striatal cell types, Mätlik et al. revealed that CAG expansion is not exclusive to MSNs but also occurs in cholinergic interneurons and cerebellar Purkinje neurons (91). It seems that CAG-repeat expansion is necessary but perhaps not sufficient to drive cell death, and that thresholds for toxicity and the rate of expansion likely vary by cell type.

Beyond neurons, single-cell and spatial genomics consistently reveal profound, cell-type-specific changes in non-neuronal cells that both respond to and amplify neuronal injury (92–97). These studies converge on a unified model of HD as a disorder of progressive cellular identity collapse driven by cell-type-specific somatic instability of the *HTT* locus and its downstream molecular consequences. The earliest pathology arises in MSNs of the striatum, where gradual CAG expansion and transcriptional dysregulation erode cellular homeostasis, triggering loss of neuronal identity. Non-neuronal populations including astrocytes, microglia, and oligodendrocytes also undergo state transitions that amplify local inflammation, metabolic stress, and circuit dysfunction (Figure 3).

## Cross-disease synthesis

Single-cell studies across neurodegenerative disorders reveal shared and disease-specific features in neuronal and glial responses. Vulnerable neuronal populations frequently include large, metabolically demanding projection neurons like RORB-expressing corticocortical neurons in AD (37), SNpc dopaminergic neurons in PD (59), Betz cells and spinal α-motor neurons in ALS (67, 78), von Economo neurons in FTD (78), and medium spiny neurons in HD (86, 90). Although these neuronal classes differ across disorders, many exhibit overlapping disease-related transcriptional signatures involving synaptic dysfunction, mitochondrial impairment, proteostasis disruption, and induction of cellular stress-response programs (37, 59–62, 76, 80, 86, 89, 90, 98). However, aspects of neuronal responses are still disease specific. Cortical neurons show largely distinct transcriptional changes in AD versus PD (64). Genetic influences on vulnerability also differ across diseases. Genes linked to PD risk variants show enriched expression in susceptible dopaminergic neuron subtypes (59), and ALS/FTD risk genes show higher expression in vulnerable corticofugal projection neurons (78, 80), although risk genes in both disorders are also expressed in non-neuronal cell types. In contrast, AD risk loci show predominant enrichment in microglia rather than neurons, with additional contributions from cerebrovascular cells (47–49, 51). In HD, selective vulnerability appears to reflect cell-type-specific somatic expansion of the mutant *HTT* allele (90).

Glial and immune cells likewise display both common and disease-specific features. Microglia adopt reactive, disease-associated states involving immune and phagocytic activation in AD, PD, ALS, and HD (41, 60, 81, 98). Astrocytes likewise transition to reactive states across disorders, characterized by loss of homeostatic gene expression and activation of stress-related pathways (45, 64, 76, 92, 94). Although glial cells in AD and PD share more differentially expressed genes than neurons, supporting the presence of recurring reactive transcriptional states, disease-specific transcriptional signatures are also apparent within glial populations (64), indicating that glial responses retain both shared and distinct features across disorders.

## Challenges and future outlook

Single-cell techniques have transformed neurodegeneration research by defining affected cell types, characterizing disease-associated states, and revealing the regulatory and spatial factors that shape these states. But key challenges remain. Isolation of whole cells from CNS tissue for scRNA-seq can distort cellular representation by enriching or depleting particular populations and can induce dissociation-associated transcriptional responses (99–102). These artifacts are substantially reduced with nuclei isolation, which avoids enzymatic digestion, enables the use of frozen postmortem CNS tissue, and better preserves the native diversity of cell types (12, 99, 103). However, snRNA-seq captures only nuclear RNA and therefore misses transcripts localized to the soma and neuronal processes, leaving key aspects of neuronal biology underrepresented. Both scRNA-seq and snRNA-seq also exhibit notable dropout events (i.e., failure to detect expressed transcripts owing to limited mRNA capture efficiency), which disproportionately affects low-abundance genes and contributes to sparsity in single-cell datasets (104, 105). Spatial transcriptomics helps address some of these limitations by profiling RNA within intact tissue, though current platforms remain expensive, low-throughput, and difficult to scale to large cohorts, and depending on the platform, they may provide lower transcriptomic coverage than dissociative approaches (105).

Spatial transcriptomics is rapidly advancing and newer high-resolution assays now achieve single-cell or even subcellular spatial readouts with improved detection efficiency, and next-generation platforms increasingly support whole-transcriptome and even isoform-resolved measurements (106). This is an important development for diseases such as ALS/FTD, where RNA splicing defects (107–111) and alternative polyadenylation changes (112–114) are central to disease pathogenesis. Several spatial platforms support joint RNA-protein detection, enabling direct comparisons between neighboring cells with and without disease-associated pathology (e.g., TDP-43 aggregates), clarifying the molecular consequences of pathological inclusions.

A major challenge for spatial and single-nucleus profiling of postmortem human CNS tissue is obtaining enough high-quality samples and profiling sufficient numbers of cells within them. Many neuronal populations of interest, such as spinal motor neurons, are intrinsically rare (75), and neurodegenerative disease further depletes the most vulnerable types, limiting their representation in postmortem datasets. Sample quality also varies widely in postmortem human CNS tissue, with factors such as RNA integrity, postmortem interval, and agonal state contributing to variability in transcriptomic measurements (115–117). Because these factors are often outside experimental control, they remain important determinants of dataset quality and key factors complicating efforts to build comprehensive and comparable human single-nucleus and spatial transcriptomic resources.

Limited temporal resolution represents another major challenge. Neurodegenerative diseases unfold over years to decades, yet most human single-cell studies sample only late pathological stages, making it difficult to distinguish early causal events from downstream or compensatory responses. Integrating datasets collected across pathological stages can, in principle, help bridge this temporal gap, but early and intermediate-stage samples remain uncommon because these studies rely largely on postmortem tissue. When staging information is available, such as the pathology-based staging in Gazestani et al. and Gabitto et al. (41, 43), it provides a framework for inferring early or intermediate states that are difficult to discern when analyzing only the most severely affected cases. Computational approaches like pseudotime analysis can provide an inferred ordering of cells along putative disease progression, offering hypotheses about molecular trajectories and potential early events (118, 119).

Model systems provide complementary opportunities to capture temporal dynamics experimentally. Genetically engineered mouse models allow in vivo analysis across defined disease stages and can reveal circuit-level or cell-cell interactions that are impossible to assess in vitro. However, even the best models only approximate human disease and vary widely in how well they recapitulate human selective vulnerability, molecular pathology, and disease pathogenesis. Human induced pluripotent stem cell– (iPSC-), organoid-, and assembloid-based systems enable the study of human-specific cell types and permit controlled perturbations, but lack the full cellular, circuit, and immune complexity of the CNS in vivo (120–122). Together, these systems can be used to provide essential mechanistic insight, yet they complement, rather than replace, postmortem human studies.

The growing use of “multiomics” in neurodegeneration research promises to link regulatory landscapes with transcriptional outputs at single-cell resolution. However, RNA and chromatin accessibility are sometimes generated in parallel rather than from the same nucleus, which can make it difficult to assign specific regulatory elements to downstream gene expression with confidence. Joint multiome assays help mitigate this limitation, although they introduce their own technical biases. As datasets generated across platforms, donors, and laboratories continue to expand, batch effects, reproducibility, standardized preprocessing, and robust cross-cohort harmonization will become increasingly important. Meta-analytic efforts further highlight the value of robust data integration, as cross-cohort analyses can reveal cellular insights that individual studies may not detect on their own.

Emerging single-cell omics technologies are expanding profiling beyond transcriptomics and chromatin accessibility. These include single-cell genome sequencing to detect somatic mutations and repeat expansions (90, 123, 124), DNA methylation to assess epigenetic remodeling (125), CUT&Tag to profile histone marks and transcription factor binding (126), chromatin conformation assays to map long-range interactions (127, 128), and proteomics to bridge discrepancies between RNA abundance and protein levels (129). Single-cell proteomics has been applied to spinal motor neurons in ALS, establishing a foundation for pathology-guided proteomic profiling at single-cell resolution (130). Extending this approach across neurodegenerative disease contexts and regions of the CNS could reveal how distinct pathological proteins reshape cellular proteomes and identify potential therapeutic targets that are only evident at the protein level. For repeat-expansion diseases, proof-of-principle work in HD has linked CAG-repeat length to transcriptomes at single-cell resolution (90); analogous approaches for other neurodegenerative disease genes, such as *C9ORF72* hexanucleotide expansions in ALS/FTD, would directly connect repeat length with transcriptional consequences, unifying genotype and molecular phenotype within individual cells.

**Figure 4:**
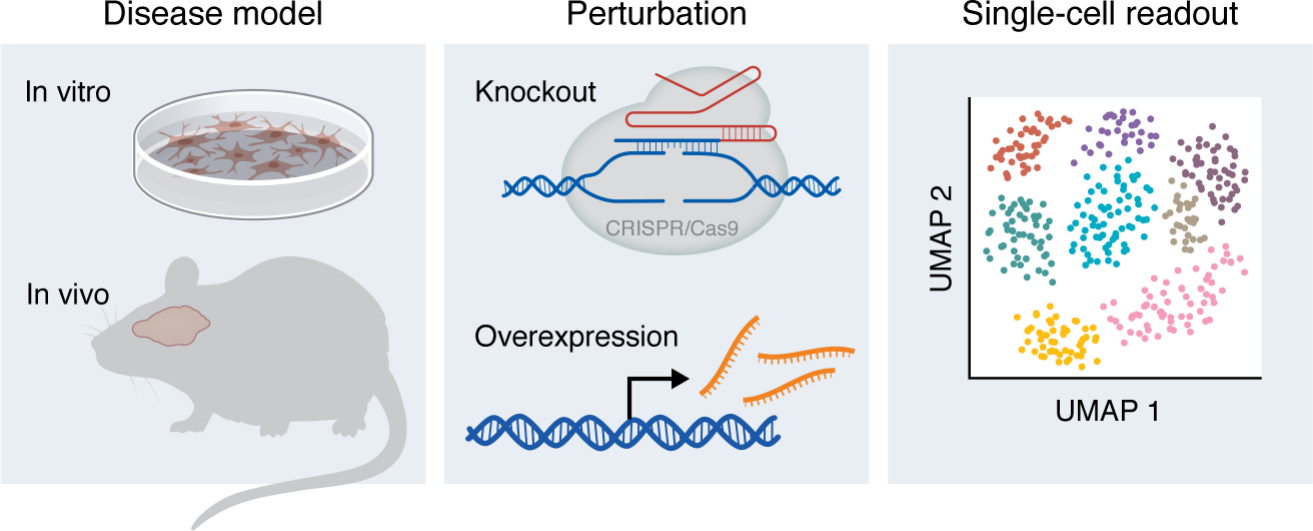
Single-cell perturbation framework to test causal regulators of vulnerability and degeneration. Left: Disease models — in vitro (e.g., iPSC-derived neurons or glia) and in vivo (e.g., gene-edited or transgenic mice) models that are relevant for a neurodegenerative disease. Middle: Perturbation — reduced expression (e.g., knockout) or increased expression (e.g., overexpression) of candidate genes or the whole genome in a pooled or arrayed format. Right: Single-cell readout — scRNA-seq, scATAC-seq, multiome, or spatial assays to quantify shifts in cell-state composition, transcription factor motif accessibility/expression, and neuronal survival due to a given perturbation. This framework links genetic/perturbational inputs to cell-state outcomes, enabling causal inference and therapeutic target selection.

Because molecular changes in disease may reflect either pathogenic or compensatory processes, functional studies are essential to pinpoint drivers of neurodegeneration. A practical path from observation to mechanism involves nominating candidate genes from descriptive datasets, perturbing them in relevant model systems, and evaluating whether the resulting cell states recapitulate or modify those seen in disease. Perturbation-based approaches (e.g., Perturb-seq and pooled overexpression screens with sc/snRNA-seq readouts) directly test whether specific genes drive or reverse disease-associated states, moving the field from description toward causality (131) (Figure 4). In one in vitro single-cell perturbation screen, Dräger et al. performed a CRISPRi CROP-seq screen in iPSC-derived microglia, showing that these cells adopt a spectrum of states resembling those observed in human brains and identifying regulators of these states (132). For example, they found that *CSF1R* knockdown reduced the proportion of cells in an *SPP1*^+^, disease-associated state (132). Recent in vivo Perturb-seq studies demonstrate that pooled CRISPR screens with single-cell readouts can be applied to the mammalian brain (133–135). Extending these strategies to neurodegenerative disease models will enable direct testing of causal mechanisms underlying neuronal selective vulnerability and degeneration to identify candidate therapeutic strategies.

## Funding support

This work is the result of NIH funding, in whole or in part, and is subject to the NIH Public Access Policy. Through acceptance of this federal funding, the NIH has been given a right to make the work publicly available in PubMed Central.

- NIH grants R35NS137159, U54NS123743, and R01AG064690.
- Live Like Lou Graduate Fellowship (to TPN).
- Target ALS (to ADG).
- SMA Foundation (to ADG).
- Stanford Graduate Fellowship (to TPN).
- Biohub – San Francisco Investigator grant (to ADG).

## Version 1 03/16/2026

Electronic publication
